# Bayesian network analysis of long-term oncologic outcomes of open, laparoscopic, and robot-assisted radical cystectomy for bladder cancer

**DOI:** 10.1097/MD.0000000000030291

**Published:** 2022-08-26

**Authors:** Lin Dong, Feng Xiaoli, Lu Ya, Wu Dan, Hu Jingwen, Liu Xun, Chen Shujin, Zhou Zhijun, Zhang Tian, Luo Hao, Yi Chuanlang, Chen Guangrong, Wang Xiaodong, Luo Gewen, Zhang Yichi, Cao Pei, Liu Yang, Wang Youliang

**Affiliations:** a Department of Urology, Pengzhou People’s Hospital, Chengdu, Sichuan, China; b Department of Respiratory and Critical Care Medicine, The First Affiliated Hospital of Chengdu Medical College, Chengdu, Sichuan, China; c ICU, Affiliated Hospital of Chengdu University of Traditional Chinese Medicine, Chengdu, Sichuan, China; d Department of Urology, Affiliated Hospital of Chengdu University, Chengdu, Sichuan, China; e Department of Anesthesiology (Operating Room), Pengzhou People’s Hospital, Chengdu, Sichuan, China; f Department of Orthopedics, Affiliated Hospital of Chengdu University, Chengdu, Sichuan, China; g Department of Gastrointestinal Surgery, Pengzhou People’s Hospital, Chengdu, Sichuan, China; h Department of Laboratory, Pengzhou People’s Hospital, Chengdu, Sichuan, China.

**Keywords:** Bayesian network analysis, bladder cancer, laparoscopic radical cystectomy, long-term oncologic outcomes, open radical cystectomy, robot-assisted radical cystectomy

## Abstract

To understand the long-term oncologic outcomes of open radical cystectomy (ORC) versus laparoscopic radical cystectomy (LRC) versus robot-assisted radical cystectomy (RARC) for bladder cancer (BCa). Therefore, we performed the conventional meta-analysis and network meta-analysis to evaluate the long-term oncologic outcomes of ORC, LRC, and RARC for BCa.

**Methods:**

A systematic search of PubMed, Embase, Cochrane Library, Medline, and Web of science was performed up until July 1, 2021. Long-term oncologic outcomes include the 5-year overall survival (OS) rate, the 5-year recurrence-free survival (RFS) rate, and the 5-year cancer specific-survival (CSS) rate. The Bayesian network analysis has been registered in PROSPERO (CRD42020208396).

**Results:**

We found that 10 articles (including 3228 patients) were included in our Bayesian network analysis. No significant differences were found between ORC, LRC, and RARC in long-term oncologic outcomes in either direct meta-analysis or network meta-analysis. Therefore, the clinical effects of 5-year OS, RFS, and CSS of RARC, LRC, and ORC are similar. But LRC may be ranked first in 5-year OS, RFS, and CSS compared to other surgical approaches by probabilistic analysis ranking via Bayesian network analysis.

**Conclusion:**

We found that there were no statistical differences in the 3 surgical approaches of RAPC, LPC, and OPC for Bca in long-term oncologic outcomes by direct meta-analysis. However, Subtle differences between these surgical approaches can be concluded that LRC may be a better surgical approach than RARC or ORC in long-term oncologic outcomes by probabilistic analysis ranking via Bayesian network analysis. Moreover, we need a large sample size and more high-quality studies to improve and verify further.

## 1. Introduction

The annual incidence of bladder cancer (BCa) accounts for about 3% of systemic malignancies.^[1]^ A variety of factors affect the occurrence and development of BCa, both protective factors and promoting factors.^[2,3]^ However, what we are talking about is that surgery is more beneficial for long-term oncological outcomes of BCa. The recognized gold standard for patients with high-risk nonmuscle-invasive and muscle-invasive BCa was open radical cystectomy (ORC) because its long-term oncological outcomes have been well-established.^[4]^ With the mature application of high-resolution image technology and the increasing learning curve of surgeons. laparoscopic (LRC) and robotic-assisted (RARC) radical cystectomy have been applied in clinical. For these 3 surgical methods, perioperative and intermediate results have been reported.^[5]^ However, the differences in long-term oncology outcomes of RARC, LRC, and ORC are unclear.

There are no statistical differences here for randomized controlled trials (RCTs) in the oncological outcomes of 3 surgical approaches for the treatment of BCa.^[6]^ The purpose of our study was to perform a network meta-analysis of 3 surgical approaches to long-term oncology outcomes (reported for at least a 5-year follow-up period).

## 2. Methods

### 2.1. Literature search and selection

The methodology involved in this meta-analysis was based on the Preferred Reporting Items for Systematic Reviews and meta-analysis (PRISMA) statement and the protocol for this systematic review and NMA has been registered in PROSPERO (CRD42020208396). The systematic literature was searched by databases such as PubMed, Embase, Cochrane Library, Medline, and Web of science. Besides, relevant journals were manually searched. We are based on the population, intervention, comparator, and outcomes (PICO) methodology. PICO was defined as follows: population consisted of patients who had clinical stage Ta-T4/N0-3/M0 BCa (P). ORC or LRC or RARC: (I) or (C). 5-year overall survival (OS) rate, 5-year recurrence-free survival (RFS) rate, and 5-year cancer specific-survival (CSS) rate (O). The search strategy was in Supplementary material 1, Supplemental Digital Content 1, http://links.lww.com/MD/H135. Search the database until July 1, 2021. Assistance strategy by manual search found as much detailed article information as possible. After reading the full text, the data were extracted. data extraction includes author, publication year, study design, age, ASA, BMI, follow-up, sample size, main observation indicators, etc.

### 2.2. Data extraction and quality evaluation

Two researchers (Lin Dong and Chen Lin) independently have reviewed the retrieved literature by the inclusion and exclusion criteria. When disagreements were encountered, the third researcher (Hu Tinghui) was required to participate in the discussion to determine whether to include. If the following inclusion criteria were met, the studies were included in the network analysis: (1) patients were diagnosed with BCa based on their pathological data. (2) Patients in each group have previously received ORC or LRC or RARC. (3) Outcome indexes should include at least one of the following, 5-year OS rate, 5-year RFS rate, and 5-year CSS rate. (4) It was limited to a randomized controlled trial or a retrospective case-control or a prospective cohort design. (5) The studies were limited to English. Any study that did not conform to the above criteria was excluded.

### 2.3. Statistical analysis

Statistical analysis was performed using Revman 5.3, Stata 12.0, Stata/MP 14.0, and ADDIS-1.16.5 software. For the meta-analysis, the heterogeneity test was *P* < .1, *I*^2^ > 50%, the random effect model was used; the heterogeneity test was *P* > .1, *I*^2^ < 50%, and the meta-analysis was performed using a fixed utility model. The combined *r*-values and 95% Confidence Interval (CI) of each study were calculated, and the characteristics of each study result were displayed on the forest map. The Begg test and Egger test were used to testing the publication bias. The *P* < .05 was considered statistically significant. For network analysis, fill in the extracted data information in the Excel table, the multiple 3-arm trials were Sorted out in a 2-arm trial format, and a net-like relationship diagram comparing multiple interventions was drawn by ADDIS-1.16.5 software. Calculate the Relative Odds Ratio and implement an inconsistency test to evaluate the closed-loop consistency in the network relationship. According to the Z test, if the lower limit of 95% CI is 1, *P* > .05, it is considered that is no inconsistency, the consistency model is used for network meta-analysis; otherwise, it is inconsistency, the inconsistency model is used for network meta-analysis. Use ADDIS-1.16.5 software: 4 Markov chain simulations, set the number of tuning iterations to 20,000, the number of simulation iterations to 50,000, and the thinning interval to 10. A close to 1 indicates that the model is satisfied with convergence; draw a rank probability map and predict the possible rank probability.

## 3. Results

### 3.1. Literature search results

**Figure 1.**
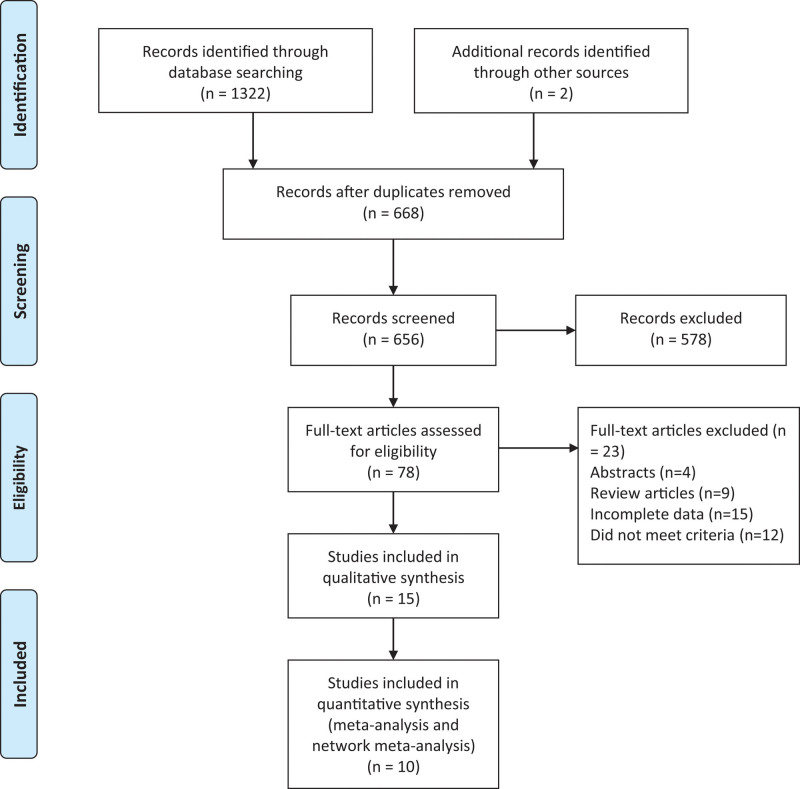
Flowchart for records selection process of the meta-analysis. (According to PRISMA template: Moher D, Liberati A, Tetzlaff J, Altman DG, The PRISMA Group (2009). Preferred Reporting Items for Systematic Reviews and Meta-Analyses: The PRISMA Statement. PLoS Med 6(7): e1000097. doi:10.1371/journal. Pmed 1000097). BCa = bladder cancer, CSS = cancer specific survival, LRC = laparoscopic radical cystectomy, ORC = open radical cystectomy, OS = overall survival, PFS = recurrence-free survival, RARC = robot-assisted radical cystectomy.

A total of 1322 articles were retrieved according to the customized search strategy and 2 additional articles. Six hundred sixty-eighth articles that were repeatedly published and cross-published were deleted. After reading the title and abstract, 578 articles were excluded. After the remaining 78 articles were searched for full text, reading, and quality assessment, 10 articles (3228 participants) were eventually included (Figure 1). The methodological quality evaluation of 10 articles^[6–15]^ included in this study can be found in Table 1 and risk bias is included in randomized controlled trials in Supplementary Figure 1, Supplemental Digital Content 2, http://links.lww.com/MD/H136.

**Table 1:** The main characteristics of included studies.

Author	Study design	Follow-up (yr)	Group	No. of participants	Age (yr)	ASA	BMI (kg/m^2^)	Male proportion (%)	Urinary diversion	Clinical stage	Pathological stage	NOS score (Max: 9)
Aboumarzouk et al (2013)	Retrospective	5	LRC	75	60 ± 7.68	N/A	27.45 ± 2.2	88	Yes		T2-T4	6
			ORC	80	62 ± 8.6		27.49 ± 3.76	82.5				
Akin et al (2013)	Prospective	5	LRC	15	62.2 ± 8.1	N/A	25.0 ± 1.1	N/A	Yes	T1-T2	T1-T2	6
			ORC	15	64.0 ± 11.9		25.1 ± 1.4					
Celen et al (2018)	Retrospective	5	LRC	35	61 (50–80)	1–3	25.34 (20.09–38.46)	88.6	Yes	T3-T4	T3-T4	6
			ORC	39	67 (48–91)		26.81 (17.31–35.03)	76.9				
Esquinas et al (2018)	Prospective	5	LRC	86	67,9 ± 9,9	2.3 ± 0.64	N/A	N/A	Yes	T1-T4	T1-T4	6
			ORC	70	65,7 ± 8,65	2.1 ± 0.58						
Faraj et al (2019)	Retrospective	5	RARC	203	73.0 (67.0–78.0)	3 (2–3)	27.4 (25.0–30.6)	82.3	N/A	Tis-T4	T2-T4	8
			ORC	278	71 (65.0–77.0)		28.1 (24.3–31.8)	82				
Gandaglia et al (2016)	Retrospective	5	RARC	138	70.0 (60.7–77.0)	1–4	26.1 (22.9–28.6)	83.3	Yes	T1-T4	Tis-T4	8
			ORC	230	70.9 (63.1–77.5)		26.0 (23.5–29.0)	83.5				
Huang et al (2021)	Retrospective	5	LRC	185	64 (58–72)	1–4	24.31 (22.05–26.26)	89.9	Yes	Ta-T4	Ta-T4	7
			ORC	185	66 (59–71)	1–3	24.22 (22.15–26.45)	88				
Bochner BH et al (2018)	RCT	5	RARC	60	66 (60–71)	≥2	N/A	85	Yes	T1-T4	T1-T4	
			ORC	58	65 (58–69)			72				
Khan et al (2019)	RCT	5	RARC	20	68 (65–74)	1–3	27.50 (24.00–31.00)	85	Yes		T0-Tis, T3-T4	
			LRC	19	71 (63–75)		26.00 (24.00–27.59)	79			T0-T3	
			ORC	20	68 (58–74)		26.99 (23.76–30.44)	90			T0-T4	
Lin et al (2014)	RCT	5	LRC	26	63.2 ± 9.1	2–3	22.0 ± 2.7	91.4	N/A	T1-T3	T1-T4	
			ORC	21	63.6 ± 8.9		22.9 ± 3.1					

ASA = American Society of Anesthesiologist, BMI = body mass index, LRC = laparoscopic radical cystectomy, N/A = not available, NOS score = Newcastle-Ottawa Scale score, ORC = open radical cystectomy, RARC = robot-assisted radical cystectomy.

### 3.2. Direct meta-analysis

The summary odds ratios (ORs) of long-term oncologic outcomes (5-year OS, RFS, and CSS rate) for every 2 direct comparisons were calculated. The network plot of the outcome indexes included in this network meta-analysis in Supplementary Figure 2, Supplemental Digital Content 3, http://links.lww.com/MD/H137 by Stata/MP 14.0. The results of the direct meta-analysis were shown by Stata 12.0 software in Supplementary Table 1, Supplemental Digital Content 4, http://links.lww.com/MD/H138. There was no statistical significance for RARC versus ORC or LRC versus ORC in 5-year OS, RFS, and CSS rate. It could not be obtained because only 1 article was included in our meta-analysis for RARC versus LRC in 5-year OS rate, 5-year RFS rate, and 5-year CSS rate.

### 3.3. Network meta-analysis

We all have used the consistency model according to Supplementary Table 2, Supplemental Digital Content 5, http://links.lww.com/MD/H139. The above results can be explained from 3 independent aspects: (a) Median is close to 0, 95% confidence interval (CrI) includes 0; (b) Median of Random Effects Standard Deviation and Inconsistency Standard Deviation is basic close to each other; (c) It was close to each other for the median of the inconsistency model and the median of the inconsistency model in random-effects standard deviation.

**Figure 2.**
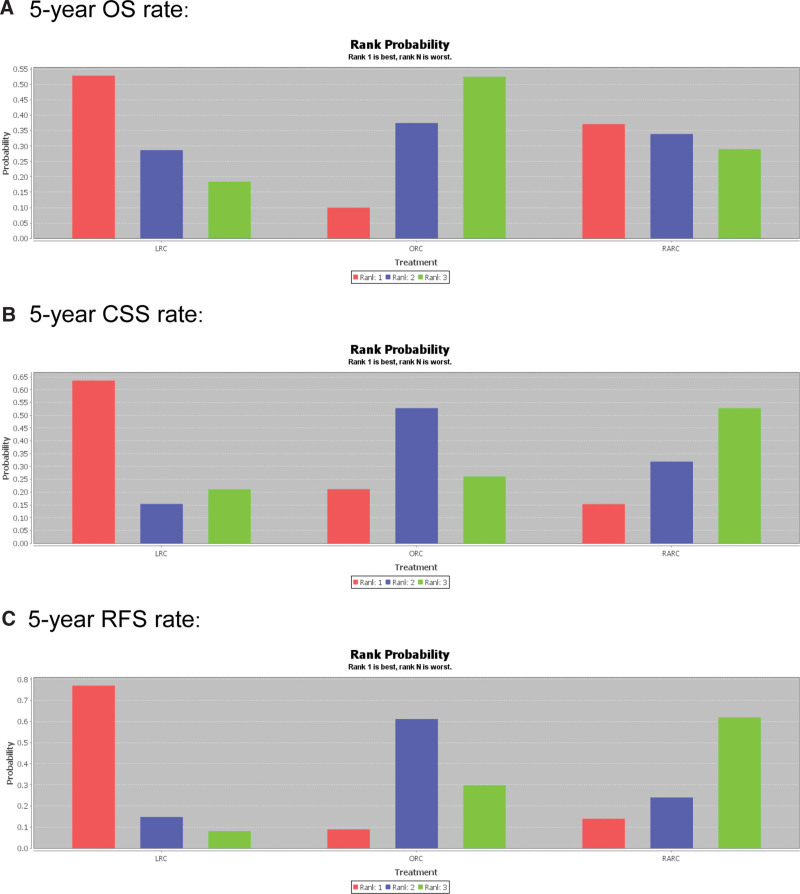
The rank probability of the 3 surgical approaches for BCa included in this meta-analysis: (a) 5-year OS rate. (b) 5-year CSS rate. (c) 5-year RFS rate. BCa = bladder cancer, CSS = cancer specific survival, OS = overall survival, PFS = recurrence free survival.

Table 2 summarizes all the studies within the multiple networks and shows the results of the mixed network comparisons. The conclusion of the confidence interval in Table 2 includes 1 shows it is concluded that there is no statistical significance. Therefore, 5-year OS, RFS, and CSS rate showed no statistical significance. But the magnitude of the probability can be shown in Figure 2 (Specific probability: Supplementary Table 3, Supplemental Digital Content 6, http://links.lww.com/MD/H140). Since Table 2 showed no statistical significance, we can compare its probability by Rank probability. Among them, we mainly look at Rank. It can be seen from Figure 2 and Supplementary Table 3, Supplemental Digital Content 6, http://links.lww.com/MD/H140 that LRC may be ranked first in 5-year OS, RFS, and CSS compared to other surgical approaches.

**Table 2:** The efficacy of 3 surgical methods according to the network meta-analysis using odds ratios (ORs) and corresponding 95% credible intervals (CrIs).

Consistent model		
5-year overall survival rate		
LRC	0.88 (0.57, 1.32)	0.96 (0.50, 1.87)
1.14 (0.76, 1.76)	ORC	1.09 (0.67, 1.88)
1.04 (0.53, 1.98)	0.92 (0.53, 1.49)	RARC
5-year cancer specific survival rate		
LRC	0.82 (0.37, 1.84)	0.75 (0.31, 1.86)
1.22 (0.54, 2.69)	ORC	0.92 (0.53, 1.54)
1.33 (0.54, 3.19)	1.08 (0.65, 1.88)	RARC
5-year recurrence free survival rate		
LRC	0.69 (0.35, 1.36)	0.61 (0.22, 1.68)
1.46 (0.74, 2.89)	ORC	0.90 (0.41, 2.00)
1.64 (0.59, 4.54)	1.11 (0.50, 2.45)	RARC

*LRC = laparoscopic radical cystectomy, ORC = open radical cystectomy, RARC = robot-assisted radical cystectomy.

### 3.4. Publication bias

We did not find any pieces of evidence about publication bias to find. The egger´s test results showed that LRC versus ORC in 5-year OS rate: *t* = 2.32, *P* = .081 (The groups with the most number of included articles: Supplementary Figure 3, Supplemental Digital Content 7, http://links.lww.com/MD/H141).

## 4. Discussion

Radical cystectomy is an effective treatment for muscular invasive and high-risk nonmuscle-invasive BCa. However, its surgical procedure is complicated, moreover, it takes a long time and has more bleed and complications.^[14]^ With the development of endoscopic technology and the increasing learning curve of surgeons. In 1992, Parra et al^[16]^ reported the first laparoscopic total cystectomy. Minimally invasive radical cystectomy has been applied to the clinic continuously. The scope of pelvic lymph node dissection under the laparoscope was the same as the open. Due to the magnifying effect of the laparoscope and the clearer field of vision, it can see the lymphatic vessels, swollen lymph nodes, Iliac vessels, obturator nerves, and other important structures to benefit from the complete removal of lymphoid tissues while avoiding neurovascular damage.^[17,18]^ Porpiglia et al^[19]^ have compared the effects of open and minimally invasive radical cystectomy on lymphadenectomy. And there was no significant difference in the number of lymph nodes dissected between the 2 groups. Some studies show that related minimally invasive radical cystectomy also has had the advantage of shorter hospital stays, reduced 90 days readmission rate, and even improved quality of life.^[20]^

Studies have shown that neoadjuvant chemotherapy also affects BCa mortality and recurrence rates. And none of our included articles did neoadjuvant chemotherapy. This is also the consistency of inclusion criteria.

So, Matsumoto et al^[21]^ found that compared with ORC, RARC, and LRC, only ORC had serious complications, and there was no difference between RARC and LRC. Although the purpose of RARC and LRC is to replicate the results of ORC, and minimally invasive is still challenging and continues to be practiced, especially laparoscope has been used clinically, it has many advantages in avoiding nerve damage and hemostasis control. For now, LRC is more widely used clinically. Khan et al^[6]^ pointed out that ORC, RARC, and LRC have similar mid-term oncology outcomes, but it is unclear when ORC, RARC, and LRC are different in long-term oncology outcomes. However, an Italian single-center confirmed LRC with extracorporeal urinary diversion, performed in centers with adequate laparoscopic experience, represents a safe procedure associated with good perioperative surgical outcomes and satisfactory long-term oncologic outcomes.^[18]^ Direct meta-analysis results from our current observational study show no significant differences in long-term oncology outcomes between ORC, RARC, and LRC. However, our network meta-analysis shows that the estimated 47%, 74%, and 64% probability that LRC is the preferred surgical approach to increase the 5-year OS rate, 5-year RFS rate, and 5-year CSS rate compared with other surgical approaches.

There were 4 limitations to the included studies. First, so far, the number of people included in RCT is relatively less. Regarding the recruitment of participants, funding problems and attitude issues are obstacles to accepting surgical procedures. The nonrandom nature of observational research makes it vulnerable to selection bias, known or unknown confounding bias. In RCT research, interventions and controls cannot achieve double blindness. The second limitation of this network meta-analysis is the small number of patients studied. Only 9 studies included 2858 patients, statistical testing may be inefficient, and conclusions must be treated with caution. The third limitation is that most of the studies included in this review come from European and American hospitals. These results may not apply to small centers and the rest of the world. The fourth limitation is the role of the surgeon, who plays an important role in the final result and in the choice of the type of technique proposed.

Because interventions and controls of RCT research cannot achieve double blindness. Therefore, future research work should focus on the improvement of RCT research methods. Recommendations for reporting RCT studies can be used as a guideline for improving research methods. Additionally, large sample size and more high-quality studies are still needed to further improve and verify.

## 5. Conclusion

We found that there were no statistical differences in the 3 surgical approaches of ORC, RARC, and LRC for BCa in long-term oncologic outcomes by direct meta-analysis. However, Subtle differences between these surgical approaches can be concluded that LRC may be better than ORC and RARC in long-term oncologic outcomes by probabilistic analysis ranking via Bayesian network analysis. Moreover, we need a large sample size and more high-quality studies to improve and verify further.

## Author contributions

**Conceptualization:** Lin Dong.

**Data curation:** Lin Dong, Wu Dan.

**Formal analysis:** Lin Dong.

**Funding acquisition:** Chen Shujin, Feng Xiaoli, Hu Jingwen, Wang Youliang.

**Investigation:** Lin Dong, Wu Dan.

**Methodology:** Chen Guangrong, Feng Xiaoli, Wang Youliang.

**Project administration:** Zhang Tian, Zhou Zhijun.

**Resources:** Hu Jingwen, Lu Ya, Luo Gewen, Zhang Yichi.

**Software:** Liu Xun, Lu Ya, Luo Hao.

**Supervision:** Chen Guangrong, Luo Gewen, Wang Xiaodong, Zhou Zhijun.

**Validation:** Yi Chuanlang, Zhang Yichi.

**Visualization:** Cao Pei, Chen Shujin, Liu Xun, Liu Yang, Wang Xiaodong.

**Writing – review & editing:** Cao Pei, Liu Yang.

## Supplementary Material


