# Supplementary material for: Bayesian network analysis of long-term oncologic outcomes of open, laparoscopic, and robot-assisted radical cystectomy for bladder cancer
Source: Medicine (Baltimore). 2022 Aug 26;101(34):e30291. doi: 10.1097/MD.0000000000030291 (PMC9410639; doi:10.1097/MD.0000000000030291)
Supplement: Supplementary file 1 [file medi-101-e30291-s001.pdf]

Supplemental material 1

The Embase datebase Search Strategy.

#1 ((ORC OR 'Open cystectomy’ OR 'Open radical cystectomy’) AND (LRC OR 'Laparoscopic cystectomy’ OR 'Laparoscopic assisted radical cystectomy’ OR 'Laparoscopic radical cystectomy’ OR 'Laparoscopic assisted cystectomy’)) OR **((**ORC OR 'Open cystectomy’ OR 'Open radical cystectomy’) AND ('Robot assisted radical cystectomy’ OR 'Robotic radical cystectomy’ OR 'Robot assisted cystectomy’ OR 'Robotic cystectomy’ OR RARC OR 'Robot assisted laparoscopic radical cystectomy’ OR 'Robot assisted laparoscopic cystectomy’)) OR ((LRC OR 'Laparoscopic cystectomy’ OR 'Laparoscopic assisted radical cystectomy’ OR 'Laparoscopic radical cystectomy’ OR 'Laparoscopic assisted cystectomy’) AND ('Robot assisted radical cystectomy’ OR 'Robotic radical cystectomy’ OR 'Robot assisted cystectomy’ OR 'Robotic cystectomy’ OR RARC OR 'Robot assisted laparoscopic radical cystectomy’ OR 'Robot assisted laparoscopic cystectomy’))

#2 'recurrence free survival’ OR 'cancer specific survival’ OR 'overall survival’ OR ' oncologic outcome’

#3 #1 AND #2
