# Supplementary figures and images for: Bayesian network analysis of long-term oncologic outcomes of open, laparoscopic, and robot-assisted radical cystectomy for bladder cancer
Source: Medicine (Baltimore). 2022 Aug 26;101(34):e30291. doi: 10.1097/MD.0000000000030291 (PMC9410639; doi:10.1097/MD.0000000000030291)

Supplementary Figure 1. Risk bias included in randomized controlled trials

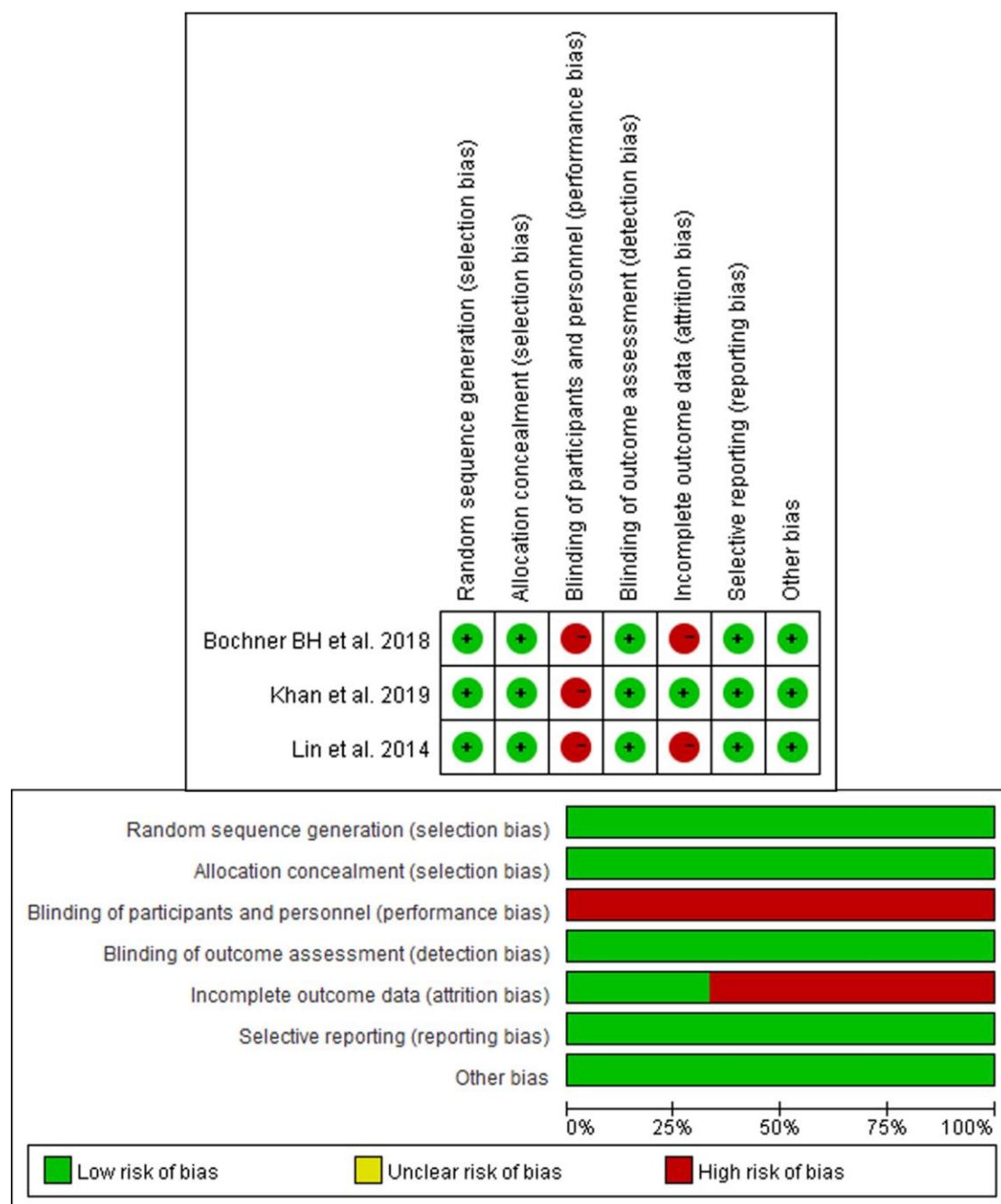

Supplement: Supplementary file 2 [file medi-101-e30291-s002.pdf]

## Supplementary Figure 2:

(a) 5-year OS rate:

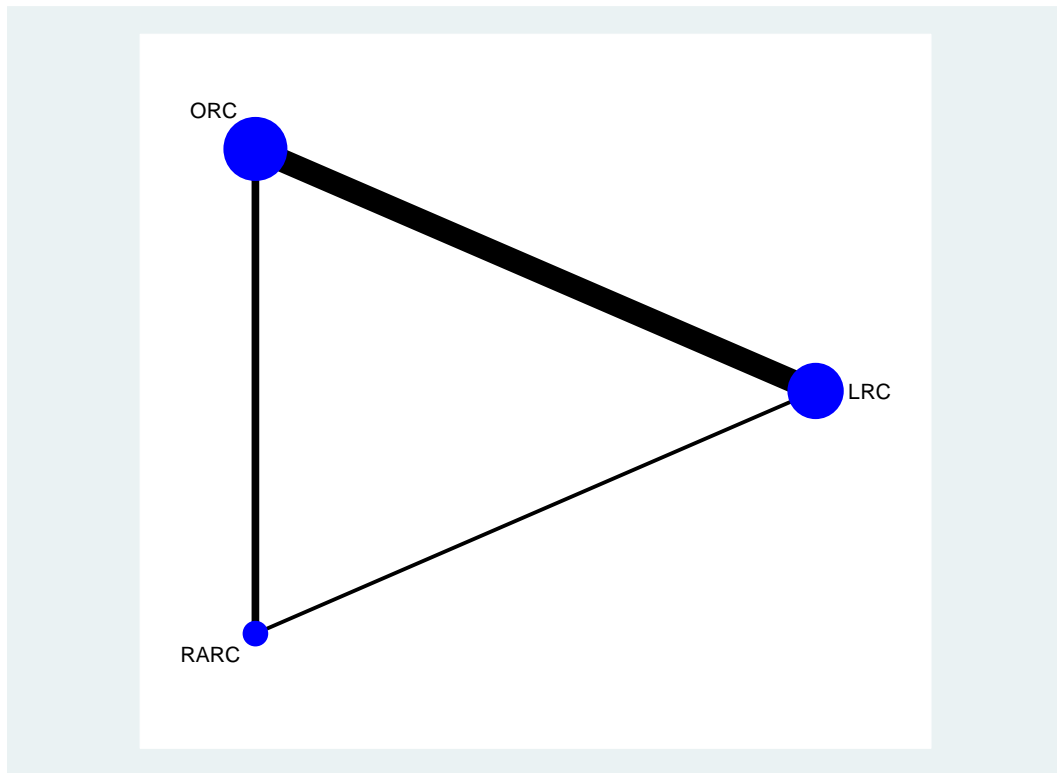

(b) 5-year CSS rate:

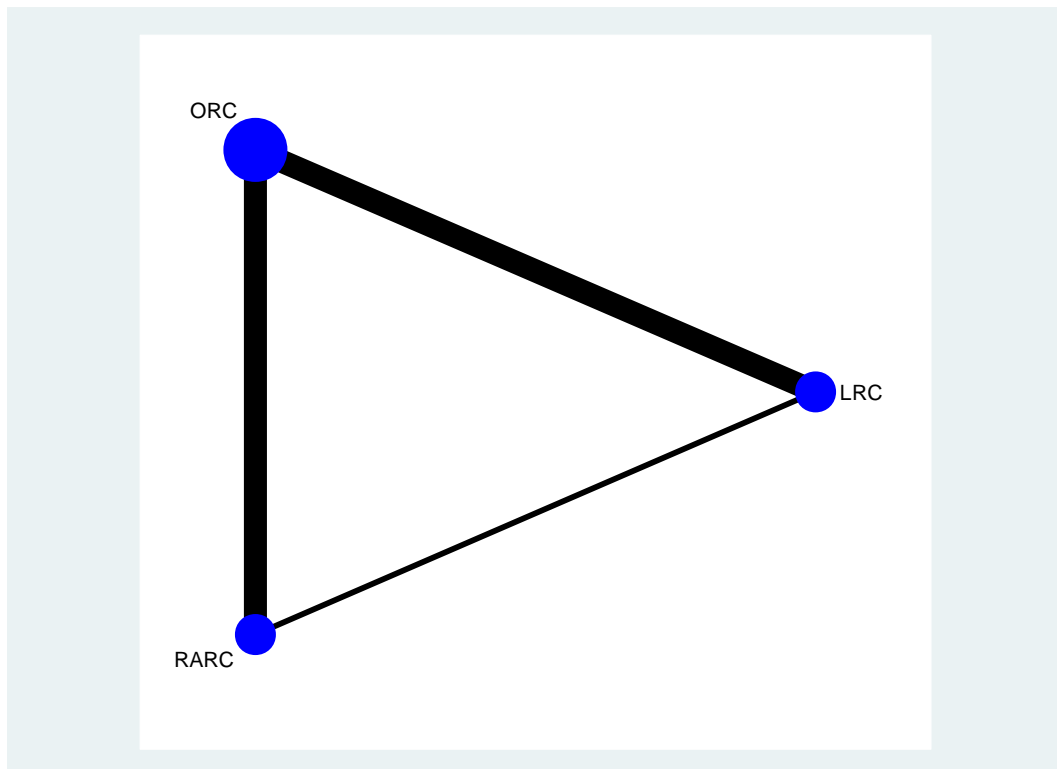

(c) 5-year RFS rate:

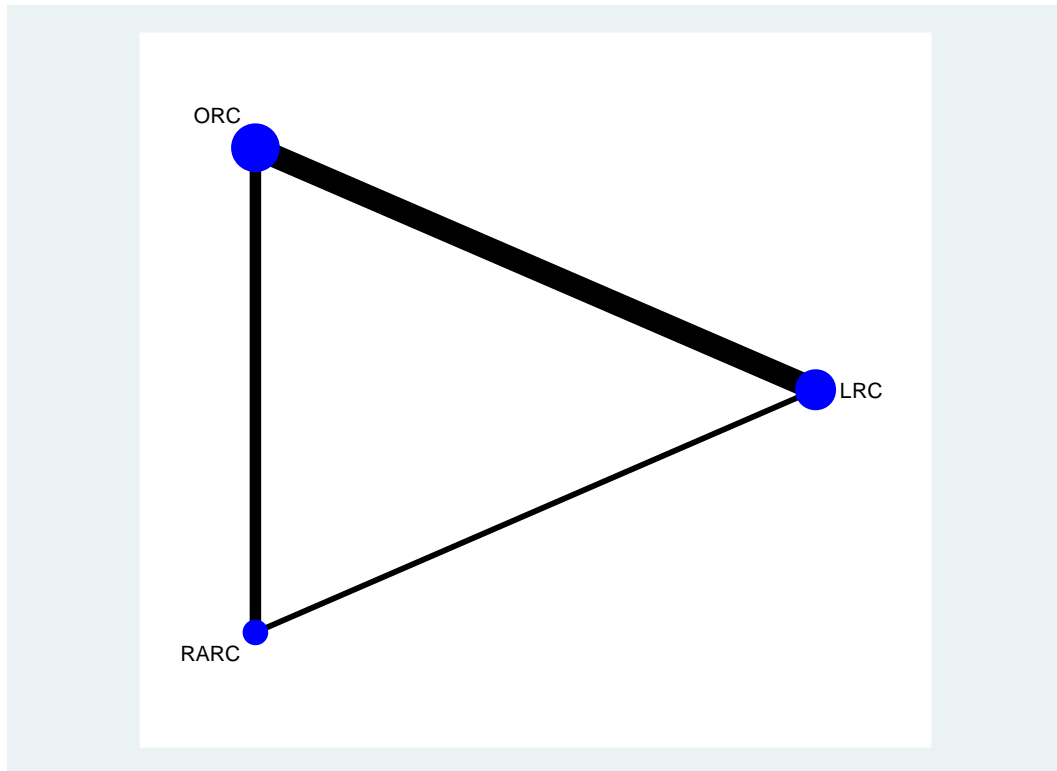

Supplement: Supplementary file 3 [file medi-101-e30291-s003.pdf]

**Supplementary Figure 3.** The egger's test results showed that LRC vs ORC in 5-year OS rate:  $t=2.32$ ,  $P=0.081$ .

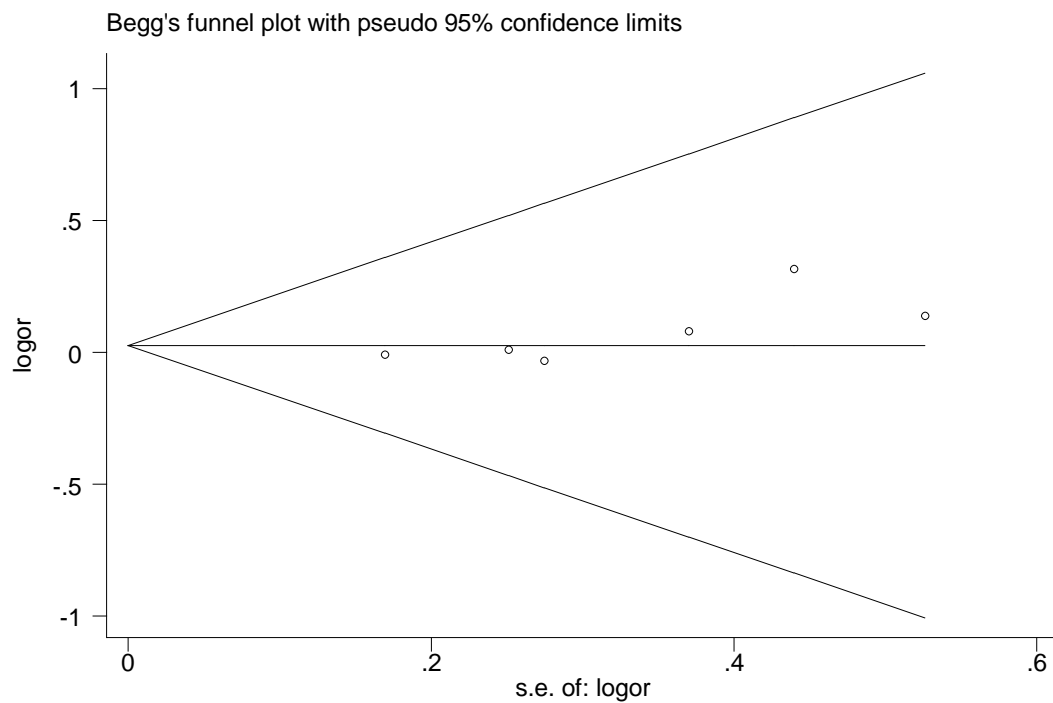

Supplement: Supplementary file 7 [file medi-101-e30291-s007.pdf]
