# Supplementary material for: Bayesian network analysis of long-term oncologic outcomes of open, laparoscopic, and robot-assisted radical cystectomy for bladder cancer
Source: Medicine (Baltimore). 2022 Aug 26;101(34):e30291. doi: 10.1097/MD.0000000000030291 (PMC9410639; doi:10.1097/MD.0000000000030291)
Supplement: Supplementary file 4 [file medi-101-e30291-s004.pdf]

Supplementary Table 1: Pair-wise meta-analyses of direct comparisons between the three surgical approaches for BCa.

End points	Direct comparisons	*I2*	PH values	OR (95% CI)	*POR* values
**5-year overall survival rate**	RARC VS ORC	close to 0%	0.795	1.04(0.78,1.38)	0.795
	RARC VS LRC	close to 0%	0.955	1.02(0.38,2.81)	0.999
	LRC VS ORC	close to 0%	0.988	1.03(0.82,1.28)	0.813
**5-year cancer specific survival rate**	RARC VS ORC	close to 0%	0.389	0.95(0.76,1.18)	0.646
	RARC VS LRC	close to 0%	0.964	1.02(0.38,2.73)	0.999
	LRC VS ORC	close to 0%	0.969	0.98(0.77,1.26)	0.895
**5-year local recurrence free survival rate**	RARC VS ORC	close to 0%	0.932	0.96(0.69,1.34)	0.806
	RARC VS LRC	close to 0%	0.686	0.81(0.30,2.20)	0.999
	LRC VS ORC	close to 0%	0.834	0.96(0.75,1.23)	0.759

*H: heterogeneity; Bca: bladder cancer; OR: odds ratio; CI: confidence interval; RARC: robot-assisted radical cystectomy, ORC: open radical cystectomy, LRC: laparoscopic radical cystectomy.
