# Supplementary material for: Bayesian network analysis of long-term oncologic outcomes of open, laparoscopic, and robot-assisted radical cystectomy for bladder cancer
Source: Medicine (Baltimore). 2022 Aug 26;101(34):e30291. doi: 10.1097/MD.0000000000030291 (PMC9410639; doi:10.1097/MD.0000000000030291)
Supplement: Supplementary file 5 [file medi-101-e30291-s005.pdf]

# Supplementary Table 2: Selection consistency model judgment table

**a**

| **Inconsistency Factors** | | |
|---|---|---|
| | **Cycle** | **Median (95% CrI)** |
| 5-year overall survival rate | LRC, ORC, RARC | -0.01 (-0.65, 0.52) |
| 5-year cancer specific survival rate | LRC, ORC, RARC | -0.04 (-0.89, 0.59) |
| 5-year recurrence free survival rate | LRC, ORC, RARC | - |
| **b** | | |
| | **Parameter** | **Median (95% CrI)** |
| 5-year overall survival rate | Random Effects Standard Deviation | 0.17 (0.01, 0.48) |
| | Inconsistency Standard Deviation | 0.25 (0.01, 0.49) |
| 5-year cancer specific survival rate | Random Effects Standard Deviation | 0.36 (0.02, 0.71) |
| | Inconsistency Standard Deviation | 0.36 (0.02, 0.72) |
| 5-year recurrence free survival rate | Random Effects Standard Deviation | 0.25 (0.02, 0.56) |
| | Inconsistency Standard Deviation | 0.29 (0.01, 0.57) |
| **c** | | |
| | **Parameter** | **Median (95% CrI)** |

| | | | |
|---|---|---|---|
| 5-year overall survival rate | consistency model | Random Effects Standard Deviation | 0.15 (0.00, 0.47) |
| | inconsistency model | | 0.17 (0.01, 0.48) |
| 5-year cancer specific survival rate | consistency model | | 0.36 (0.07, 0.71) |
| | inconsistency model | | 0.36 (0.02, 0.71) |
| 5-year recurrence free survival rate | consistency model | | 0.25 (0.00, 0.56) |
| | inconsistency model | | 0.25 (0.02, 0.56) |
