# Supplementary material for: Bayesian network analysis of long-term oncologic outcomes of open, laparoscopic, and robot-assisted radical cystectomy for bladder cancer
Source: Medicine (Baltimore). 2022 Aug 26;101(34):e30291. doi: 10.1097/MD.0000000000030291 (PMC9410639; doi:10.1097/MD.0000000000030291)
Supplement: Supplementary file 6 [file medi-101-e30291-s006.pdf]

Supplementary Table 3: Rank probability: the specific probability of Figure 1a-c

(a)

Surgical approach	Rank 1	Rank 2	Rank 3
LRC	0.53	0.29	0.18
ORC	0.1	0.37	0.53
RARC	0.37	0.34	0.29

(b)

Surgical approach	Rank 1	Rank 2	Rank 3
LRC	0.64	0.15	0.21
ORC	0.21	0.53	0.26
RARC	0.15	0.32	0.53

(c)

Surgical approach	Rank 1	Rank 2	Rank 3
LRC	0.77	0.15	0.08
ORC	0.09	0.61	0.3
RARC	0.14	0.24	0.62
